# Optical and Structural Properties of Co^2+^-Doped CsPbI_3_ Nanocrystals Embedded in Borosilicate Glass

**DOI:** 10.3390/nano16100580

**Published:** 2026-05-08

**Authors:** Wilson A. Silva, Éder V. Guimarães, Klever A. S. Costa, Nataly S. Moura, José F. Condeles, Raquel A. Domingues, Ricardo S. Silva

**Affiliations:** 1 Instituto de Ciências Exatas, Naturais e Educação (ICENE), Departamento de Física, Universidade Federal do Triângulo Mineiro, Uberaba 38025-180, MG, Brazil; 2 Instituto de Ciência e Tecnologia (ICT), Universidade Federal de São Paulo, São Jose dos Campos 13083-970, SP, Brazil; radomingues@unifesp.br

**Keywords:** CsPbI_3_ nanocrystals, borosilicate glass, Co^2+^ doping, tetrahedral crystal field phase stabilization, chromaticity tuning

## Abstract

Co^2+^-doped CsPbI_3_ nanocrystals (NCs) (CsPbI_3_:xCo, x = 0, 5, and 10 mol%) were synthesized in situ within a borosilicate glass matrix by the fusion method followed by controlled thermal treatment at 500 °C for 6–24 h. Transmission electron microscopy images showed quasi-spherical NCs with mean diameters of 4.9–7.1 nm. Energy-dispersive X-ray spectroscopy suggested cobalt incorporation within the nanocrystalline regions. X-ray diffraction patterns confirmed the exclusive stabilization of the cubic α-phase across all compositions, with systematic lattice contraction from a = 6.321 Å to a = 6.301 Å with increasing Co content, consistent with preferential B-site substitution of Pb^2+^ by Co^2+^. Transmittance measurements confirmed macroscopic optical transparency of all glass-NC composites after thermal treatment. The crystal field theory and Tanabe–Sugano analysis for d^7^ ions in tetrahedral (Td) symmetry yielded Δ = 5032 cm^−1^ and B = 725 cm^−1^ in the as-prepared state, evolving to Δ = 4428 cm^−1^ and B = 805 cm^−1^ after thermal treatment, confirming Td Co^2+^ coordination and significant metal–iodide covalency. CIE 1931 chromaticity analysis revealed tunable emission from deep-red coordinates to near-white-light regions, demonstrating potential for LED and single-material WLED phosphor applications. Long-term photoluminescence measurements demonstrated full preservation of α-phase excitonic emission after approximately 365 days under ambient conditions, establishing the robust phase stability of CsPbI_3_:xCo NCs embedded in borosilicate glass.

## 1. Introduction

All-inorganic halide perovskite nanocrystals (NCs) (CsPbX_3_, X = Cl, Br, I) have attracted considerable attention in recent decades due to their interesting optical properties and potential for technological applications in devices such as solar cells, light-emitting diodes, lasers, and photodetectors [1,2,3,4,5,6]. Among these materials, CsPbI_3_ stands out because of its direct bandgap near 1.7 eV in the visible–near-infrared spectral region, making it a strong candidate for photovoltaic and light-emitting applications [7,8]. However, the cubic α-phase of CsPbI_3_, which is responsible for its excellent optical properties, is metastable at room temperature and spontaneously converts into the non-perovskite orthorhombic δ-phase under ambient conditions [8,9]. This yellow δ-phase presents a wide bandgap of ~2.8 eV and poor optoelectronic performance, which severely limits the practical use of CsPbI_3_ [9,10]. In the last decade, different stabilization strategies have been explored, including quantum confinement, compositional engineering, surface passivation, and partial ionic substitution at the B-site [11,12,13,14,15,16].

The doping of CsPbX_3_ NCs with transition-metal (TM) ions has been explored as an approach to simultaneously modulate their structural and optical properties [17]. Among the available TM dopants, Co^2+^ ions (3d^7^, high-spin) are particularly relevant due to their intense intra-3d optical transitions, whose energies and intensities are governed by crystal-field interactions and are therefore sensitive to local coordination geometry, ligand-field strength, and metal–ligand covalency [17]. This behavior has been extensively documented in chalcogenide-based NCs systems and glass–NC composites, encompassing Bi_2−x_Co_x_S_3_, Pb_1−x_Co_X_S, Zn_1−x_Co_x_O, and Zn_1−x_Co_x_Te hosts, where Co^2+^ ions serve simultaneously as functional dopants and as spectroscopic probes of local structure [18,19,20,21,22]. The role of Co^2+^ in halide perovskite matrices, however, remains comparatively limited, particularly within fully inorganic environments where dopant segregation and phase instability represent important constraints [23,24].

The synthesis of CsPbX_3_ NCs within glass matrices has emerged as an effective route to circumvent the phase instability and environmental sensitivity inherent to bulk perovskite films, affording excellent control over NC size, high thermal stability, and strong resistance to moisture-induced degradation [25,26,27,28,29]. Near the glass transition temperature (Tg), short-range atomic mobility is sufficient to drive diffusion-limited nucleation and growth of NCs with controllable size distributions, while the rigid vitreous network suppresses bulk devitrification and macroscopic phase segregation [25,26,27]. Under these nanoconfinement conditions, the cubic α-CsPbI_3_ phase can be stabilized with well-defined excitonic features and photoluminescence efficiency [26,30].

Borosilicate glasses are particularly well suited for hosting iodide perovskite NCs, owing to their low hygroscopicity, high thermal and chemical stability, and chemical compatibility with halide precursors [25,26,28]. The borosilicate network also provides a stable confinement environment in which transition-metal dopants incorporated at the B-site of the perovskite NCs can adopt tetrahedral (Td) coordination geometries, as reported for Co^2+^ ions in chalcogenide glass–NCl composites [18,20,28,29,30,31,32]. The crystal-field-driven optical transitions of Co^2+^ ions in Td symmetry offer direct spectroscopic access to local coordination geometry, ligand-field strength, and metal–ligand covalency. Beyond structural confinement, the borosilicate matrix serves multiple active functional roles: it provides a physical barrier against moisture-induced degradation owing to its low hygroscopicity and chemical inertness toward halide species; it controls NC size and spatial distribution through diffusion-limited growth near Tg; and it prevents macroscopic ionic migration and dopant segregation, thereby enabling long-term preservation of the optically active α-CsPbI_3_ phase under ambient conditions [25,26,29].

In this work, we report the optical and structural properties of Co^2+^-doped CsPbI_3_ NCs (CsPbI_3_:xCo, with x = 0, 5, and 10 mol%) synthesized in situ within a borosilicate glass matrix by the fusion method followed by controlled thermal treatment. The present study advances the field in several respects. First, this is, to the best of our knowledge, the first systematic investigation of Co^2+^-doped CsPbI_3_ NCs grown in situ within a borosilicate glass matrix, combining compositional tunability with intrinsic environmental protection in a single solid-state platform. Second, the Tanabe–Sugano crystal-field analysis applied here provides, for the first time, quantitative ligand-field parameters (Δ, B, β) for Co^2+^ in a CsPbI_3_ halide perovskite environment and tracks their evolution as a function of thermal treatment, offering direct spectroscopic insight into the modification of Co^2+^–iodide covalency during α-phase stabilization. Third, the long-term stability of the α-phase excitonic emission under ambient conditions is demonstrated over a timescale of approximately one year, establishing the robustness of the glass-confined NC architecture for photonic applications. X-ray diffraction (XRD), transmission electron microscopy (TEM), energy-dispersive X-ray spectroscopy (EDX), and differential thermal analysis (DTA) were employed to investigate the structural, morphological, and thermal stability of the NCs. Transmittance, optical absorption (OA), and photoluminescence (PL) spectroscopy were used to probe the optical response of the CsPbI_3_ host lattice and the electronic states introduced by Co^2+^ doping. The crystal field theory (CFT) and Tanabe–Sugano analysis for d^7^ ions in Td symmetry were applied to determine the coordination environment and ligand-field parameters of Co^2+^ upon B-site substitution and thermal treatment.

## 2. Materials and Methods

### 2.1. Glass Preparation and In Situ Nanocrystal Growth

A borosilicate glass matrix with nominal molar composition 40SiO_2_–39B_2_O_3_–1Al_2_O_3_–5Cs_2_O–5PbI_2_–10NaI (mol%) was employed as the host medium for the in situ growth of CsPbI_3_:xCo nanocrystals (NCs). In this notation, *x* denotes the molar percentage of Co^2+^ substituting Pb^2+^ in the perovskite lattice. Samples were prepared with x = 0, 5, and 10 mol%, corresponding to nominal compositions CsPb_1−x_Co_x_I_3_. For clarity, the samples are labeled as CsPbI_3_:0Co, CsPbI_3_:5Co, and CsPbI_3_:10Co NCs, respectively. The glass matrix was prepared from the following reagents, all purchased from Sigma-Aldrich (St. Louis, MO, USA) at purities of ≥99.0%: silicon dioxide (SiO_2_), boron trioxide (B_2_O_3_), aluminum oxide (Al_2_O_3_), cesium oxide (Cs_2_O), lead(II) iodide (PbI_2_), and sodium iodide (NaI, anhydrous). Cobalt was introduced as cobalt (II) oxide (CoO), providing Co^2+^ ions directly in a form chemically compatible with the oxide glass network and avoiding the introduction of additional anions that could disrupt the halide stoichiometry of the system.

Sample preparation followed the fusion method. Stoichiometric amounts of the precursor powders were thoroughly homogenized in an agate mortar and transferred to an alumina crucible. The batch was melted at 1300 °C for 20 min in an electric furnace under a carbon-assisted reducing atmosphere provided by graphite carbon placed within the furnace chamber. This procedure, standard in our laboratory for iodide-containing glass compositions, serves to suppress halide volatilization at high temperature and to stabilize the Co^2+^ oxidation state during melting, inhibiting oxidation to Co^3+^. The resulting melt was rapidly quenched onto a preheated stainless-steel plate to suppress uncontrolled crystallization, yielding optically transparent, homogeneous glass. The samples used for all optical measurements had a uniform thickness of approximately 2.0 mm.

Thermal treatments were subsequently carried out at 500 °C for 6, 10, and 24 h to induce diffusion-driven nucleation and growth of CsPb_1−x_Co_x_I_3_ NCs within the glass matrix. This temperature lies near the glass transition temperature (Tg) of the borosilicate host, as confirmed by differential thermal analysis (Section 3.4), where short-range atomic mobility of Pb^2+^, Cs^+^, I^−^, and Co^2+^ species is sufficient to drive confined crystallization within the rigid vitreous network, enabling controlled NC growth without macroscopic phase segregation or bulk devitrification.

### 2.2. Structural and Spectroscopic Characterization

The formation, size, shape, and spatial dispersion of the CsPbI_3_:xCo NCs within the glass matrix were examined by transmission electron microscopy (TEM) using a JEOL JEM-2100 instrument (JEOL Ltd., Tokyo, Japan) operated at 200 kV. Mean particle diameters and size distributions were obtained from statistical analysis of N = 50 NCs per sample condition using the ImageJ software [33]. Energy-dispersive X-ray spectroscopy (EDX), integrated with the TEM column, was used to evaluate the local elemental composition and to verify cobalt incorporation into the nanocrystalline regions.

The crystalline structure of the CsPbI_3_:xCo NCs was identified by X-ray diffraction (XRD) using a Shimadzu XRD-6000 diffractometer (Shimadzu, Kyoto, Japan) with Cu Kα_1_ monochromatic radiation (λ = 1.54056 Å). Diffraction profiles were collected over the angular range of 10–60° (2θ), with a step size of 0.02° and a dwell time of 2 s per step. Phase attribution was performed using the JCPDS database.

Thermal properties were investigated by differential thermal analysis (DTA) using a Shimadzu DTA-50 system (Shimadzu, Kyoto, Japan). Measurements were performed from room temperature up to 700 °C at a heating rate of 20 °C/min in air, using 30 mg of powdered glass samples placed in alumina crucibles (5 mm in diameter and height) with alumina as an inert reference material. The glass transition temperature (Tg) and crystallization temperature (Tc) were determined from the DTA curves.

Optical absorption (OA) spectra were recorded in the 190–900 nm range using a Shimadzu UV-2600 double-beam UV–Vis spectrophotometer (Shimadzu, Kyoto, Japan), with the bare borosilicate glass as reference. Transmittance spectra were recorded over the same spectral range using the same instrument, with air as reference, to evaluate the optical transparency of the glass-NC composites as a function of thermal treatment and Co^2+^ content.

Photoluminescence (PL) spectra were obtained under continuous-wave laser excitation at 355 nm (≈3.49 eV), with an incident power of 12 mW focused to a spot of approximately 200 µm on the sample surface. The emitted light was collected and dispersed by an Avantes multichannel spectrometer (Avantes, Apeldoom, The Netherlands) operating in the 200–1100 nm range. CIE 1931 chromaticity coordinates were computed from corrected emission spectra to quantify emission color and its evolution with thermal treatments. Long-term photoluminescence stability was evaluated by recording normalized PL spectra of the thermally treated samples (500 °C, 24 h) on the day of preparation (day 1) and after approximately 365 days of storage under ambient laboratory conditions (room temperature, ambient atmosphere, no humidity control).

All measurements were performed at room temperature.

## 3. Results and Discussion

### 3.1. Nanocrystal Morphology and Size Distribution

**Figure 1:**
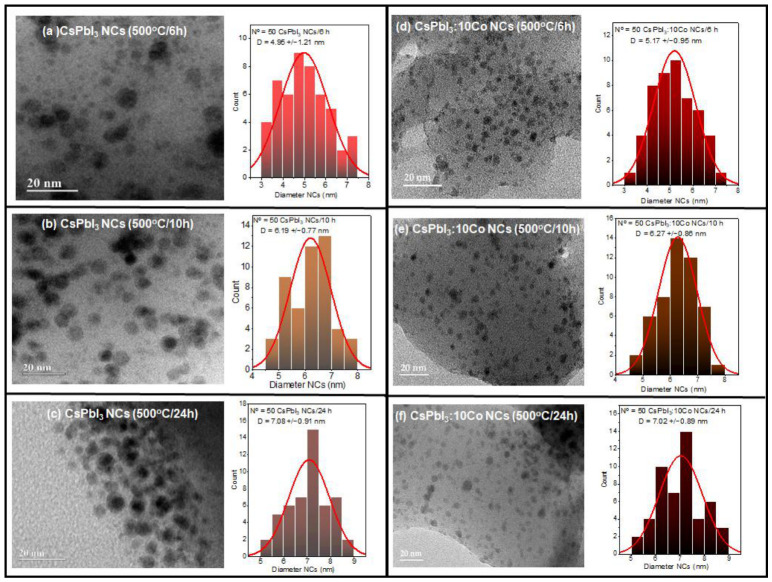
TEM micrographs and corresponding particle size distribution histograms with Gaussian fits of CsPbI_3_ (**a**–**c**) and CsPbI_3_:10Co (**d**–**f**) NCs grown in a borosilicate glass matrix after thermal treatment at 500 °C for 6 h (**a**,**d**), 10 h (**b**,**e**), and 24 h (**c**,**f**). Mean diameters are 4.95 ± 1.21, 6.19 ± 0.77, and 7.08 ± 0.91 nm for CsPbI_3_, and 5.17 ± 0.95, 6.27 ± 0.86, and 7.02 ± 0.89 nm for CsPbI_3_:10Co, respectively. Statistical analysis based on N = 50 NCs per condition. The systematic size increase with annealing time and the comparable dimensions between undoped and Co^2+^-doped samples are consistent with diffusion-mediated growth under glass nanoconfinement.

Figure 1 shows TEM micrographs and corresponding particle size distribution histograms with Gaussian fits of CsPbI_3_ (a–c) and CsPbI_3_:10Co (d–f) NCs grown in the borosilicate glass matrix after thermal treatment at 500 °C for 6 h (a, d), 10 h (b, e), and 24 h (c, f). In all conditions, discrete nanometric domains are clearly resolved within the amorphous matrix, exhibiting quasi-spherical morphology. The well-defined contrast between the NCs and the surrounding glass host is consistent with the higher electron density of the CsPbI_3_ phase relative to the borosilicate matrix, confirming successful in situ crystallization [25,26,29].

Statistical analysis of N = 50 nanocrystals per condition revealed a systematic increase in mean diameter for both compositions. For CsPbI_3_, the size evolved from 4.95 ± 1.21 nm (6 h) to 6.19 ± 0.77 nm (10 h) and 7.08 ± 0.91 nm (24 h). For CsPbI_3_:10Co, the values increased from 5.17 ± 0.95 nm (6 h) to 6.27 ± 0.86 nm (10 h) and 7.02 ± 0.89 nm (24 h). Overall, this corresponds to a total size increment of approximately 43% across the annealing window for both compositions. The size distributions are unimodal at all three annealing times, following Gaussian profiles, indicating that NC growth proceeds without detectable secondary nucleation events [25,29]. The mean diameters of CsPbI_3_ and CsPbI_3_:10Co are statistically comparable at all annealing times, confirming that Co^2+^ incorporation at the concentrations studied does not measurably affect NC growth kinetics or final particle size. The progressive narrowing of the standard deviation with annealing time was observed both for CsPbI_3_, from ±1.21 nm (6 h) to ±0.91 nm (24 h), and for CsPbI_3_:10Co, from ±0.95 nm (6 h) to ±0.89 nm (24 h). This behavior is consistent with a growth regime dominated by the gradual incorporation of diffusing ionic species (Pb^2+^, Cs^+^, I^−^) into pre-existing nuclei, rather than by renewed burst nucleation [29].

The monotonic enlargement of NCs with annealing time demonstrates that thermal treatment at 500 °C sustains continued mass transport at the NC–glass interface, driven by the residual atomic mobility available near Tg [25,26,34], as confirmed by the DTA results presented in Section 3.4. The unimodal character of the distributions at all annealing times is indicative of a growth regime dominated by the progressive incorporation of diffusing ionic species into pre-existing nuclei, rather than by renewed burst nucleation [29]. Even after 24 h of annealing, the NCs remain confined below 10 nm, showing that the rigid borosilicate network effectively restricts long-range ionic diffusion and limits excessive coarsening [25,26,29]. This size range places all samples within or near the quantum confinement regime of CsPbI_3_, whose exciton Bohr radius is estimated at ~3–4 nm [30], and is directly relevant to the optical properties discussed in Section 3.5.

### 3.2. Structural Analysis by X-Ray Diffraction

**Figure 2:**
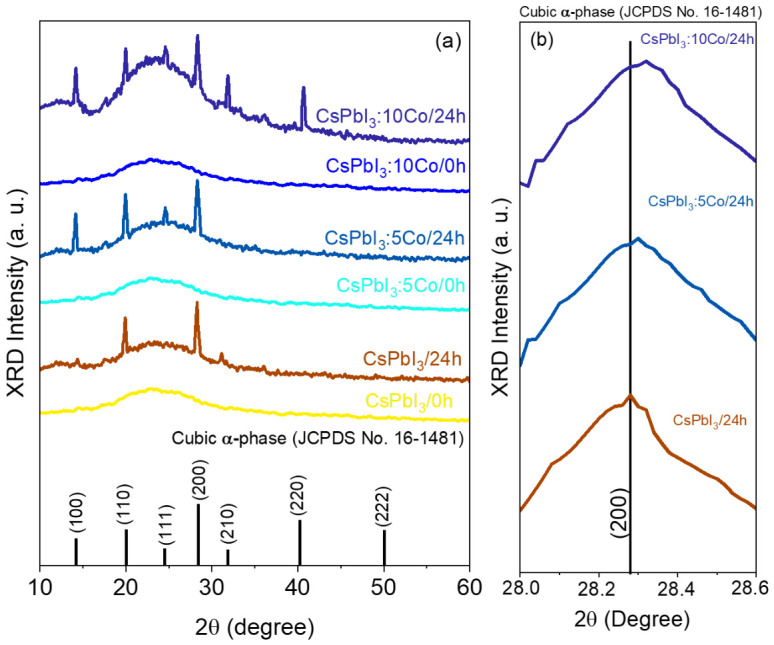
XRD patterns of CsPbI_3_, CsPbI_3_:5Co, and CsPbI_3_:10Co NCs grown in a borosilicate glass matrix after thermal treatment at 500 °C for (0 h/24 h). (**a**) Diffraction profiles showing Bragg reflections indexed exclusively to the cubic α-CsPbI_3_ phase (space group Pm3^−^m, JCPDS No. 16-1481), superimposed on the broad amorphous halo of the glass host. (**b**) Enlarged view of the (200) reflection showing the systematic shift to greater 2θ values with increasing Co^2+^ content, evidencing B-site lattice contraction consistent with substitutional incorporation of Co^2+^.

Figure 2a shows the XRD patterns of the as-prepared (0 h) borosilicate glass prior to any thermal treatment, confirming that the melt-quenching procedure yields a fully amorphous matrix, as evidenced by the broad amorphous halo centered near 22–25° with no resolved Bragg reflections attributable to any crystalline phase, including α-CsPbI_3_, δ-CsPbI_3_, CoI_2_, or cobalt oxide secondary phases. Figure 2a shows the XRD patterns of CsPbI_3_, CsPbI_3_:5Co, and CsPbI_3_:10Co NCs grown in the borosilicate glass matrix after thermal treatment at 500 °C for 24 h, corresponding to the condition of maximum NC size established in Section 3.1. All diffractograms display a broad halo centered near 22–25°, characteristic of the amorphous borosilicate glass host, superimposed by well-resolved Bragg reflections indexed exclusively to the cubic α-CsPbI_3_ phase (space group Pm3^−^m, JCPDS No. 16-1481) [9,13,25,26]. The diffraction peaks observed at approximately 14.2°, 20.1°, 24.5°, 28.3°, 31.8°, 40.5°, and 50.1° are assigned to the (100), (110), (111), (200), (210), (220), and (222) crystallographic planes, respectively. No additional reflections attributable to the orthorhombic δ-CsPbI_3_ phase, cobalt iodide (CoI_2_), or cobalt oxide secondary phases are detected in any of the patterns, confirming that the thermal treatment protocol selectively stabilizes the cubic α-phase across all compositions and that Co^2+^ incorporation does not induce phase separation [9,10,23,27].

Figure 2b shows a magnified view of the (200) reflection for the three compositions. A systematic shift of the peak maximum toward higher 2θ values is observed with increasing cobalt content, from 28.27° (d_200_ ≈ 0.3161 nm) in CsPbI_3_ to approximately 28.29° (d_200_ ≈ 0.3154 nm) in CsPbI_3_:5Co and 28.32° (d_200_ ≈ 0.3151 nm) in CsPbI_3_:10Co. According to Bragg’s law (nλ = 2d sinθ), a shift toward higher diffraction angles at constant wavelength implies a reduction in the interplanar spacing d_200_ and, consequently, a contraction of the cubic unit cell parameter. To quantify this contraction, the cubic lattice parameter a and unit cell volume V were extracted from the (200) reflection using Bragg’s law and the interplanar spacing relation for the cubic system (d_200_ = a/√(h^2^ + k^2^ + l^2^)). The results are summarized in Table 1. The monotonic decrease in both a and V with increasing Co^2+^ content confirms progressive lattice contraction consistent with Vegard’s law behavior reported for analogous transition-metal-doped lead halide perovskite systems.

The observed lattice contraction is consistent with the partial substitution of Pb^2+^ (ionic radius ≈ 1.19 Å in 6-fold coordination) by Co^2+^ ions (ionic radius ≈ 0.72 Å in tetrahedral coordination) at the B-site of the ABX_3_ perovskite structure. Upon B-site incorporation, Co^2+^ adopts a tetrahedral coordination geometry (Td) rather than the octahedral environment (Oh) of Pb^2+^, inducing local distortion of the cubic network and the observed unit cell contraction [20,27]. The systematic reduction in lattice parameter with increasing x is consistent with Vegard’s law behavior reported for analogous transition-metal-doped lead halide perovskite systems [23,27]. The local structural distortion introduced by Co^2+^ in Td geometry is expected to modify the B–X bonding framework and contribute to the thermodynamic stabilization of the cubic α-phase [10,12,14,35].

### 3.3. Compositional Analysis by TEM–EDX

**Figure 3:**
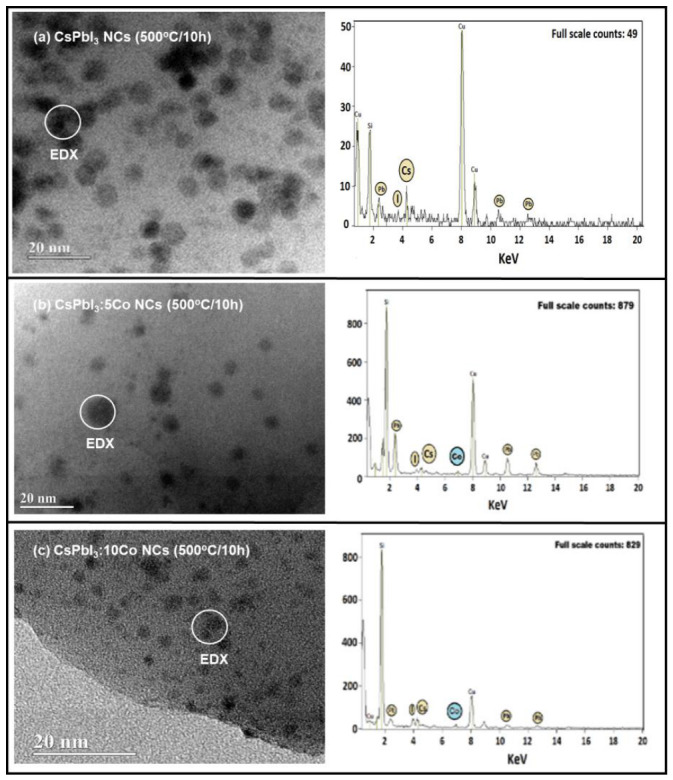
TEM micrographs and corresponding EDX spectra of CsPbI_3_, CsPbI_3_:5Co, and CsPbI_3_:10Co NCs grown in a borosilicate glass matrix after thermal treatment at 500 °C for 10 h: (**a**) x = 0, (**b**) x = 0.05, and (**c**) x = 0.10 mol%. The Co Kα signal at ~6.9 keV is absent in the undoped sample and detected in both Co^2+^-doped compositions, confirming cobalt incorporation within the nanocrystalline regions without evidence of cobalt-rich secondary phases.

Figure 3 shows representative TEM micrographs and corresponding EDX spectra of CsPbI_3_, CsPbI_3_:5Co, and CsPbI_3_:10Co NCs subjected to thermal treatment at 500 °C for 10 h. The micrographs confirm the formation of NCs dispersed within the borosilicate glass matrix, displaying quasi-spherical morphology and mean diameters on the order of ~7 nm [25,26], in agreement with the size distributions established in Section 3.1 for this annealing condition. Across all three compositions, the NC morphology and spatial distribution remain essentially unchanged, showing that Co^2+^ incorporation at concentrations up to 0.10 mol% does not measurably affect nucleation density, growth kinetics, or particle morphology under the selected thermal treatment parameters [27,29].

The EDX spectrum of the undoped sample (Figure 3a) shows emission lines assigned to Cs (L_α_ ≈ 4.3 keV), Pb (M_α_ ≈ 2.3 keV; L_α_ ≈ 10.5 keV; L_β_ ≈ 12.6 keV), and I (L_α_ ≈ 3.9 keV), confirming the chemical identity of the cubic α-CsPbI_3_ perovskite phase [25]. An additional signal at ~1.7 keV (Si K_α_) is attributed to the surrounding borosilicate glass matrix, inevitably sampled by the electron beam given the ~7 nm NC dimensions relative to the probe interaction volume. The prominent Cu Kα signal at ~8 keV originates from the TEM support grid [18,19].

For the Co^2+^-doped samples (Figure 3b,c), an additional emission feature appears at approximately 6.9 keV, attributed to the Co K_α_ transition and highlighted by the blue marker in both spectra. This signal is absent in the undoped reference, confirming cobalt incorporation within the nanocrystalline regions [19,20,27]. The dominant Si K_α_ signal observed in the doped sample spectra reflects the unavoidable contribution of the borosilicate matrix to the interaction volume, a common limitation in EDX characterization of sub-10 nm NCs embedded in oxide glass matrices [18,29]. No emission lines assignable to CoI_2_ or cobalt oxide secondary phases are detected in any spectrum, consistent with the XRD results of Section 3.2 [20,23].

EDX analysis in TEM mode yields spatially averaged compositional information from a region substantially larger than an individual NC and does not independently resolve crystallographic site occupancy or the formal oxidation state of cobalt. Taken together with the XRD results of Section 3.2, specifically the systematic (200) peak shift indicative of B-site lattice contraction, the EDX data are consistent with the incorporation of Co^2+^ within the cubic α-CsPbI_3_ nanocrystalline regions most plausibly at B-sites of the perovskite structure, rather than segregation into a separate cobalt-rich phase [20,23,27].

### 3.4. Thermal Characterization by Differential Thermal Analysis

**Figure 4:**
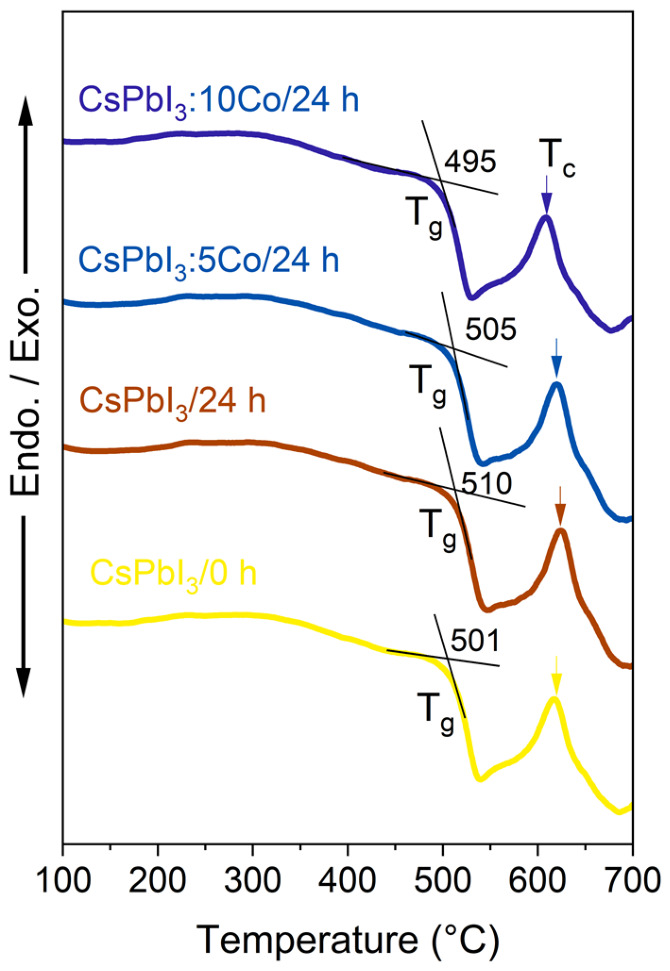
Differential thermal analysis (DTA) curves of borosilicate glass samples containing CsPbI_3_:xCo (x = 0, 5, and 10) NC precursors under different thermal-treatment conditions. The curves reveal glass transition temperatures (Tg) in the range of 495–510 °C and a crystallization (Tc) near 615 °C. The dashed vertical line indicates the annealing temperature used for NC growth (500 °C), located close to Tg, where short-range ionic diffusion is activated while avoiding bulk devitrification. The slight shift of Tg with increasing Co content suggests a modifier effect of Co ions on the borosilicate glass network.

The thermal properties of the glass-NC composites were investigated by DTA to establish the relationship between the selected thermal treatment temperature and the characteristic temperatures of the borosilicate host. The DTA curves presented in Figure 4 reveal glass transition temperatures of Tg = 501 °C (CsPbI_3_, 0 h), 510 °C (CsPbI_3_, 24 h), 505 °C (CsPbI_3_:5Co, 24 h), and 495 °C (CsPbI_3_:10Co, 24 h), and a crystallization temperature Tc ≈ 615 °C common to all compositions. These data confirm that the thermal treatment temperature of 500 °C lies in the immediate vicinity of Tg across all compositions, providing the short-range ionic mobility of Cs^+^, Pb^2+^, I^−^, and Co^2+^ species required for diffusion-driven NC nucleation and growth, while remaining well below Tc, ensuring that macroscopic devitrification does not occur. The slight systematic decrease of Tg with increasing Co^2+^ content (510 → 505 → 495 °C) suggests that Co^2+^ acts as a network modifier within the borosilicate matrix, locally disrupting the vitreous network and enhancing short-range ionic mobility. This is consistent with the role of transition-metal ions as network modifiers in oxide glass systems, where their incorporation into non-bridging sites weakens the connectivity of the vitreous network [28]. The thermal stability window defined by Tg and Tc, spanning approximately 105–120 °C across all compositions, provides a well-defined processing range within which controlled NC growth can be achieved without macroscopic phase separation or bulk crystallization of the glass host.

### 3.5. Optical Properties and Crystal-Field Analysis

**Figure 5:**
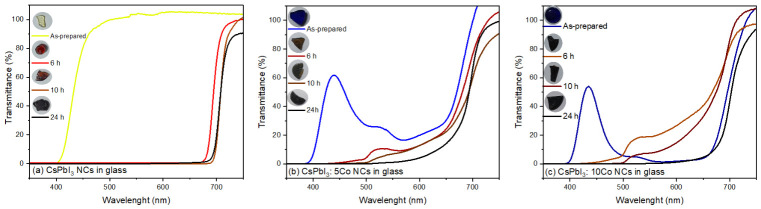
UV–Vis transmittance spectra and digital photographs of CsPbI_3_:xCo (x = 0, 5, and 10) borosilicate glass-NC samples after thermal treatment at 500 °C for different annealing times. All samples remain macroscopically transparent after in situ nanocrystal growth. The undoped compositions exhibit higher visible transmittance, whereas Co-doped samples show reduced transmittance and progressive blue coloration due to Co^2+^ crystal-field absorption. Variations in the absorption edge with annealing time are associated with the formation and growth of embedded CsPbI_3_ NCs.

Figure 5 shows the transmittance spectra of CsPbI_3_, CsPbI_3_:5Co, and CsPbI_3_:10Co glass-NC composites after thermal treatment at 500 °C for 0, 6, 10, and 24 h, together with photographs of representative samples illustrating their visual appearance. In the as-prepared condition, the undoped CsPbI_3_ glass (Figure 5a) exhibits high transmittance across the entire visible range (~100%), consistent with the fully amorphous, NC-free matrix confirmed by XRD (Section 3.2), and appears colorless-yellow in transmitted light. Upon thermal treatment, the transmittance edge progressively shifts toward shorter wavelengths as α-CsPbI_3_ NCs nucleate and grow, reflecting the development of band-edge absorption in the red spectral region [25,26]. All annealed samples retain macroscopic optical transparency with no haze or turbidity, confirming that NC growth remains confined to the nanometric regime and that bulk phase segregation does not occur [25,26,29]. The Co^2+^-doped samples in the as-prepared condition (Figure 5b,c) exhibit pronounced absorption bands in the 400–600 nm range, directly visible as the characteristic deep blue coloration of the glass discs shown in the inset photographs, attributed to Co^2+^ intra-3d crystal-field transitions in tetrahedral coordination [18,20]. Upon thermal treatment, these bands are progressively attenuated as the growing α-CsPbI_3_ NC population dominates the optical response, and the samples evolve toward darker appearances consistent with the red band-edge absorption of the α-phase [25,26].

**Figure 6:**
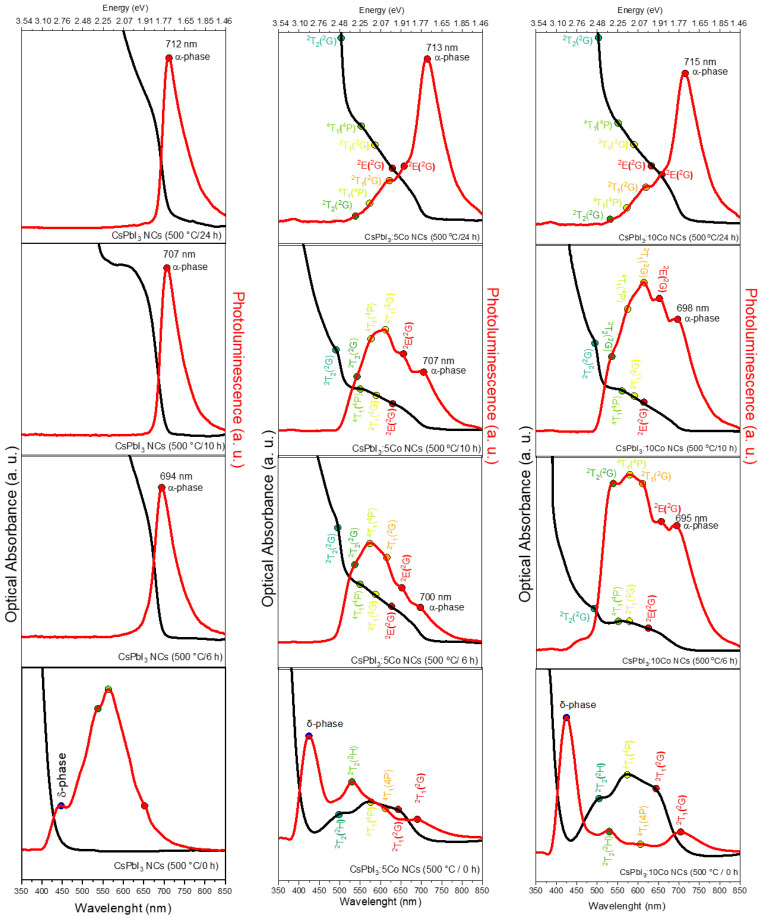
OA (black curves) and PL (red curves) spectra of CsPbI_3_, CsPbI_3_:5Co, and CsPbI_3_:10Co NCs grown in a borosilicate glass matrix after thermal treatment at 500 °C for 0, 6, 10, and 24 h. In the undoped sample, the absorption edge and band-edge excitonic emission progressively redshift with annealing time, reflecting NC growth, relaxation of quantum confinement, and progressive stabilization of the cubic α-CsPbI_3_ phase. In the Co^2+^-doped samples, additional absorption bands assigned to the ^4^A_2_(^4^F) → ^2^T_2_(^2^H), ^4^A_2_(^4^F) → ^4^T_1_(^4^P), and ^4^A_2_(^4^F) → ^2^T_1_(^2^G) crystal-field transitions of tetrahedrally coordinated Co^2+^ are resolved in the as-prepared condition (0 h), evolving to four bands assigned to ^4^A_2_(^4^F) → ^2^E(^2^G), ^4^A_2_(^4^F) → ^4^T_1_(^4^P), ^4^A_2_(^4^F) → ^2^T_1_(^2^G), and ^4^A_2_(^4^F) → ^2^T_2_(^2^G) after thermal treatment.

Figure 6 shows the OA and PL spectra of CsPbI_3_, CsPbI_3_:5Co, and CsPbI_3_:10Co NCs grown in the borosilicate glass matrix after thermal treatment at 500 °C for 0, 6, 10, and 24 h. The spectral evolution gives simultaneous access to two distinct electronic subsystems: the extended band states of the CsPbI_3_ host, which govern the excitonic response, and the localized intra-3d levels of Co^2+^, which reflect the crystal-field environment at the dopant site [7,9,17].

In the as-prepared condition (0 h), the OA and PL spectra of the undoped CsPbI_3_ sample are characteristic of the δ-phase. The absorption onset is located near 430 nm, consistent with the wide optical bandgap of the δ-phase (~2.8 eV) [9,14], and the PL spectrum shows a broad emission band centered near ~565 nm, attributed to radiative recombination within δ-phase nanocrystallites [9,25]. The presence of δ-phase emission in the as-prepared condition is consistent with the XRD result of Section 3.2, which confirms the fully amorphous nature of the glass matrix prior to thermal treatment; the broad PL emission at 0 h is therefore attributed to crystalline δ-phase clusters formed during quenching that are below the XRD detection limit. In the Co^2+^-doped samples at 0 h, three absorption bands are clearly resolved in the OA spectra, superimposed on the δ-phase background, and their intensities scale with cobalt concentration. Concurrently, the δ-phase PL emission also increases in intensity with Co^2+^ content. This behavior is tentatively consistent with a defect-passivation mechanism, in which Co^2+^ ions may occupy structural defect sites within the δ-phase nanocrystallites, improving local crystallographic order and thereby enhancing radiative recombination efficiency [18,19].

The transition from δ-phase emission in the as-prepared condition to α-phase excitonic emission upon annealing reflects the thermally activated reorganization of ionic species within the glass matrix near Tg, as established by DTA (Section 3.4) [25,26,29]. In the as-prepared condition, rapid quenching from 1300 °C kinetically traps the system in the δ-phase, which is the thermodynamically stable polymorph of CsPbI_3_ at ambient temperature and pressure [8,12]. Upon thermal treatment at 500 °C, sufficient short-range ionic mobility is restored to allow diffusion-driven reorganization of Cs^+^, Pb^2+^, and I^−^ species [25,26]. Under nanoconfinement, the rigid borosilicate network suppresses long-range ionic diffusion that would otherwise promote bulk δ-phase formation, while the high surface-to-volume ratio of the confined NCs lowers the thermodynamic barrier for α-phase stabilization [25,29]. The role of Co^2+^ in this process may involve local lattice distortion at the B-site that contributes to α-phase stabilization. However, this contribution is described as possible rather than confirmed in the absence of direct mechanistic probes [31,32].

Upon thermal treatment at 500 °C for 6 and 10 h, the OA and PL spectra show systematic evolution in all compositions. In the undoped CsPbI_3_ sample, the absorption edge progressively shifts toward longer wavelengths and a narrow, high-intensity excitonic PL band assigned to band-edge recombination of the cubic α-CsPbI_3_ phase is stabilized at 694 nm (6 h) and 707 nm (10 h) [9,13,25]. This spectral evolution directly reflects NC growth and progressive stabilization of the cubic α-phase established in Section 3.1, Section 3.2 and Section 3.3. Given that the exciton Bohr radius of CsPbI_3_ is estimated at ~3–4 nm and the NCs span 6.3–8.4 nm, intermediate quantum confinement is operative during the early annealing stages [30]. As the NCs grow, confinement effects are progressively relaxed, contributing to the observed redshift of the excitonic emission. In the Co^2+^-doped samples at 6–10 h, the α-phase excitonic emission appears at 700–707 nm for CsPbI_3_:5Co, where PL intensity is attenuated relative to the undoped reference, and at 695–698 nm for CsPbI_3_:10Co, where PL intensity increases considerably. The non-monotonic dependence of excitonic PL intensity on Co^2+^ concentration at these intermediate annealing times, attenuation at x = 5 mol% and enhancement at x = 10 mol%, suggests that the net optical effect of Co^2+^ incorporation arises from the competition between defect passivation and the introduction of Co^2+^-related non-radiative recombination channels. At x = 5 mol%, the dopant concentration may be insufficient to passivate the majority of non-radiative defect sites, while simultaneously introducing new recombination pathways, resulting in net PL attenuation. At x = 10 mol%, passivation of a larger fraction of defect sites may dominate, producing net PL enhancement.

After thermal treatment at 500 °C for 24 h, the excitonic PL emission stabilizes at 712 nm for CsPbI_3_, 713 nm for CsPbI_3_:5Co, and 715 nm for CsPbI_3_:10Co, with PL maxima near ~1.74 eV across all compositions, confirming that Co^2+^ incorporation at the concentrations studied preserves the fundamental optical response of the cubic α-CsPbI_3_ host [9,25,30]. The enhanced PL intensity observed for CsPbI_3_:10Co at intermediate annealing times may be tentatively related to modifications in the quantum confinement regime induced by Co^2+^ incorporation at the B-site. Concurrently, the Co^2+^ absorption bands, clearly resolved at earlier annealing stages, become progressively masked in the 24 h spectra, attributed to the growth of α-CsPbI_3_ NCs whose absorption edge shifts toward ~690 nm with increasing annealing time, reflecting relaxation of quantum confinement [25,30].

**Figure 7:**
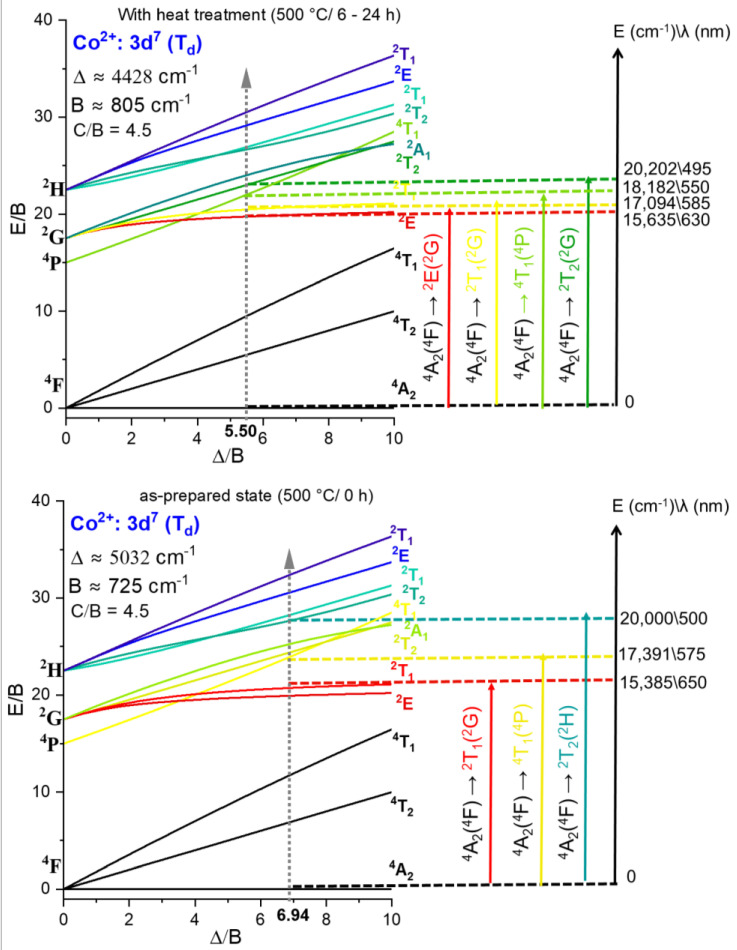
Tanabe–Sugano diagrams for Co^2+^ (3d^7^, Td symmetry, C/B = 4.5) in the as-prepared state (500 °C, 0 h; lower panel) and after thermal treatment (500 °C, 6–24 h; upper panel). In the as-prepared state, the crystal-field parameters Δ = 5032 cm^−1^, B = 725 cm^−1^, and Δ/B = 6.94 are extracted from the ^4^A_2_(^4^F) → ^2^T_2_(^2^H), ^4^A_2_(^4^F) → ^4^T_1_(^4^P), and ^4^A_2_(^4^F) → ^2^T_1_(^2^G) transitions. After thermal treatment, four transitions are resolved and the parameters evolve to Δ = 4428 cm^−1^, B = 805 cm^−1^, and Δ/B = 5.50, reflecting weakening of the crystal-field splitting and partial reduction of metal–ligand covalency associated with lattice reorganization upon α-phase stabilization.

The three absorption bands observed at 0 h are characteristic of Co^2+^ (3d^7^) ions in tetrahedral coordination (Td), consistent with the local reordering of Co^2+^ upon B-site substitution established in Section 3.2 and with the well-documented preference of Co^2+^ for Td symmetry in halide environments [17,18,36]. Based on the Tanabe–Sugano analysis presented in Figure 7 (lower panel), these bands are assigned as follows: the feature at 15,385 cm^−1^ (650 nm) is attributed to the spin-forbidden ^4^A_2_(^4^F) → ^2^T_2_(^2^H) transition; the band at 17,391 cm^−1^ (575 nm) corresponds to the spin-allowed ^4^A_2_(^4^F) → ^4^T_1_(^4^P) transition, consistent with its comparatively greater oscillator strength [17,21,36]; and the higher-energy feature at 20,000 cm^−1^ (500 nm) is assigned to the spin-forbidden ^4^A_2_(^4^F) → ^2^T_1_(^2^G) transition. The coexistence of one spin-allowed and two spin-forbidden transitions is consistent with a high-spin d^7^ configuration in a weak Td ligand field, as expected for Co^2+^ in iodide-rich coordination [17,28,36].

Following thermal treatment (6–24 h), four resolved Co^2+^ absorption bands are identified in the OA spectra of the doped samples. Based on the Tanabe–Sugano diagram in Figure 7 (upper panel), these bands are assigned to the transitions ^4^A_2_(^4^F) → ^2^E(^2^G) at 15,635 cm^−1^ (630 nm), ^4^A_2_(^4^F) → ^4^T_1_(^4^P) at 17,094 cm^−1^ (585 nm), ^4^A_2_(^4^F) → ^2^T_1_(^2^G) at 18,182 cm^−1^ (550 nm), and ^4^A_2_(^4^F) → ^2^T_2_(^2^G) at 20,202 cm^−1^ (495 nm). The collective blueshift of all transitions relative to the as-prepared state reflects modifications in the Co^2+^ coordination environment associated with lattice reorganization during α-phase stabilization.

The crystal field theory and Tanabe–Sugano analysis for a d^7^ ion in Td symmetry (Figure 7) yielded, for the as-prepared state (0 h), Δ = 5032 cm^−1^, B = 725 cm^−1^, Δ/B = 6.94, and C/B = 4.5. The extracted Δ and B parameters should be interpreted as effective ligand-field descriptors of the average Co^2+^ coordination environment rather than exact unique structural values, given the distribution of local environments expected in a glass-confined nanocrystalline system. This Δ/B ratio places the system within the high-spin domain, confirming the thermodynamic stability of the ^4^A_2_ ground state and the absence of spin crossover toward a low-spin configuration [17]. The nephelauxetic ratio β = B/B_0_ = 725/1120 ≈ 0.65, taking the free-ion Racah parameter B_0_ = 1120 cm^−1^ for Co^2+^ [36], reflects significant expansion of the Co^2+^ 3d electron cloud due to covalent delocalization into iodide ligand orbitals, consistent with the high polarizability of I^−^ and its capacity for pronounced metal–ligand orbital overlap [31,32,36,37,38].

After thermal treatment (6–24 h), the crystal-field parameters evolve to Δ = 4428 cm^−1^, B = 805 cm^−1^, Δ/B = 5.50, and β = 0.72. Two correlated changes underlie this evolution. The decrease in Δ (5032 → 4428 cm^−1^) reflects a weakening of the crystal-field splitting experienced by Co^2+^, consistent with a slight increase in the average Co–I bond distance as the surrounding lattice reorganizes upon α-phase stabilization [17,28,36]. The increase in B (725 → 805 cm^−1^), evidenced by the rise in β (0.65 → 0.72), indicates a partial reduction of metal–ligand covalency and a contraction of the effective 3d orbital radius, interpreted as a relaxation of Co–I bond strain as the perovskite lattice approaches its equilibrium cubic structure [18,27,38]. Throughout this evolution, the system remains in the high-spin regime, confirming the preservation of the ^4^A_2_ ground state. The characteristic optical absorption bands and Tanabe–Sugano analysis are fully consistent with Co^2+^ species in tetrahedral coordination environments, with no spectroscopic evidence of Co^3+^ contributions, whose d^6^ configuration would produce a fundamentally different pattern of crystal-field transitions in the blue-green spectral region.

**Figure 8:**
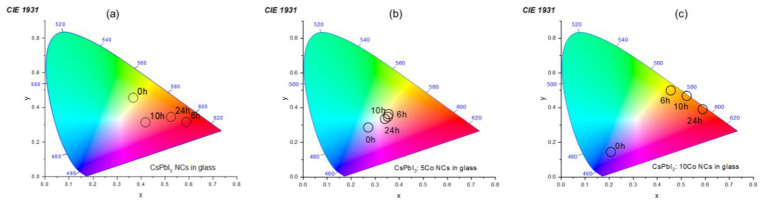
CIE 1931 chromaticity coordinates calculated from the photoluminescence spectra of CsPbI_3_:xCo (x = 0 (**a**), 5 (**b**), and 10 (**c**)) NCs embedded in borosilicate glass under different annealing times at 500 °C. The undoped sample shows a progressive shift from yellow-orange to deep-red emission with increasing thermal-treatment time, associated with the δ-to-α phase transformation of CsPbI_3_. Co-doped samples exhibit broader chromaticity tunability due to the combined contribution of Co^2+^ crystal-field transitions and perovskite excitonic emission, including coordinates approaching the white-light region at intermediate annealing times. These results demonstrate thermally controlled and dopant-assisted color modulation in glass–perovskite NCs.

CIE 1931 chromaticity coordinates were calculated from the photoluminescence spectra for all compositions and annealing conditions and are presented in Figure 8. The undoped CsPbI_3_ NCs exhibit a progressive evolution of emission chromaticity from the yellow-orange region (0 h, δ-phase emission near 565 nm) toward deep red coordinates upon thermal treatment (6–24 h), consistent with α-CsPbI_3_ band-edge excitonic emission near 712 nm, positioning these samples as candidates for solid-state red-emitting LED components [6,8,12,18]. Notably, the CsPbI_3_:5Co sample displays chromaticity coordinates at intermediate annealing times (6 h and 10 h) that migrate toward the white-light region of the CIE 1931 diagram, in the vicinity of the achromatic point (~x = 0.33, y = 0.33). This behavior arises from the simultaneous contribution of the Co^2+^ intra-3d crystal-field emission bands and the evolving α-phase excitonic emission, whose spectral overlap produces a broadband emission profile spanning the visible range, a highly desirable characteristic for single-material WLED phosphor applications [31,32,33]. The CsPbI_3_:10Co sample follows a broader chromaticity trajectory, from the blue-violet region at 0 h to deep red after 24 h annealing, further demonstrating the tunability of emission chromaticity achievable through combined Co^2+^ doping and thermal treatment control [18,31,33].

**Figure 9:**
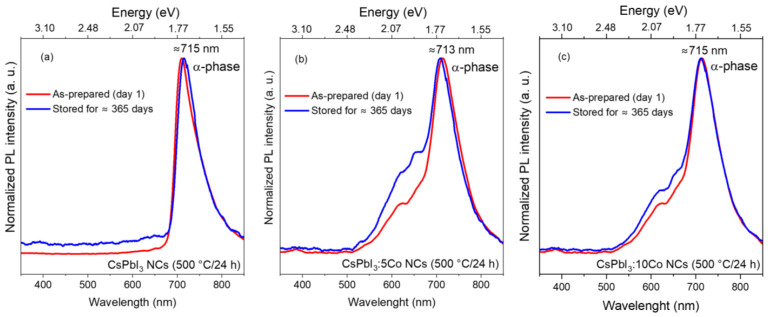
Normalized photoluminescence (PL) spectra of CsPbI_3_:xCo (x = 0 (**a**), 5 (**b**), and 10 (**c**)) NCs embedded in borosilicate glass after thermal treatment at 500 °C for 24 h, comparing the freshly prepared samples (day 1) with those stored under ambient laboratory conditions for approximately 365 days. All compositions preserve the characteristic α-CsPbI_3_ excitonic emission centered near 713–715 nm, with no significant peak shift or spectral broadening after long-term aging. The results demonstrate the excellent temporal stability of the optically active α-phase enabled by glass encapsulation, even in the presence of Co^2+^ doping.

The long-term photoluminescence stability of the thermally treated samples (500 °C, 24 h) was evaluated by comparing normalized PL spectra recorded on day 1 and after approximately 365 days of storage under ambient laboratory conditions. As shown in Figure 9a, the undoped CsPbI_3_ NCs retain the α-phase excitonic emission band centered at ~715 nm after one year of ambient storage. No measurable shift in peak position, spectral broadening, or δ-phase emission features were detected. This constitutes direct experimental evidence that the borosilicate glass matrix effectively suppresses the α → δ phase conversion under ambient conditions [6,8,18,25,29]. The Co^2+^-doped samples similarly preserve the α-phase excitonic emission at ~713 nm (CsPbI_3_:5Co, Figure 9b) and ~715 nm (CsPbI_3_:10Co, Figure 9c) after ≈365 days, confirming that Co^2+^ incorporation at concentrations up to 10 mol% does not compromise the long-term phase stability of the glass-confined nanocrystals. A subtle evolution of the spectral background in the 500–650 nm region is observed in the doped samples after prolonged storage, tentatively attributed to a slow redistribution of Co^2+^ coordination environments within the stabilized perovskite lattice; however, this does not affect the α-phase excitonic emission position or profile [31,32,38]. The OA, PL, and crystal-field data show that the optical and structural properties of glass-confined CsPbI_3_:xCo NCs are governed by the coexistence of delocalized excitonic states of the α-perovskite lattice and localized 3d crystal-field excitations of Co^2+^ [17,18,38].

## 4. Conclusions

CsPbI_3_:xCo (x = 0, 5, and 10 mol%) NCs were synthesized in situ within a borosilicate glass matrix by the fusion method followed by controlled thermal treatment at 500 °C for 6–24 h. TEM images showed quasi-spherical NCs with mean diameters increasing from 4.95 to 7.08 nm, consistent with diffusion-mediated growth under glass nanoconfinement. XRD patterns confirmed exclusive stabilization of the cubic α-phase, with systematic lattice contraction from a = 6.321 Å (V = 252.7 Å^3^) to a = 6.301 Å (V = 250.3 Å^3^) with increasing Co content, consistent with preferential B-site incorporation of Co^2+^. EDX suggested cobalt presence within the nanocrystalline regions. Differential thermal analysis confirmed that 500 °C lies near Tg (495–510 °C) and well below Tc ≈ 615 °C, establishing the mechanistic basis for controlled NC growth within the rigid borosilicate network. Transmittance measurements confirmed macroscopic optical transparency of all glass-NC composites after thermal treatment. The crystal field theory and Tanabe–Sugano analysis yielded Δ = 5032 cm^−1^, B = 725 cm^−1^, and β ≈ 0.65 in the as-prepared state, evolving to Δ = 4428 cm^−1^, B = 805 cm^−1^, and β ≈ 0.72 after thermal treatment, confirming tetrahedral Co^2+^ coordination and significant metal–iodide covalency. CIE 1931 analysis revealed tunable emission chromaticity from deep-red coordinates to near-white-light regions for CsPbI_3_:5Co, demonstrating potential for red-emitting LED and single-material WLED phosphor applications. Long-term photoluminescence measurements demonstrated full preservation of α-phase excitonic emission after ≈365 days under ambient conditions. These results establish CsPbI_3_:xCo NCs embedded in borosilicate glass as a robust, long-term stable, and chromatically tunable platform for photonic applications.

## Figures and Tables

**Table 1:** Lattice parameters extracted from the (200) XRD reflection for CsPbI_3_:xCo NCs after thermal treatment at 500 °C for 24 h.

Sample	2θ_(200)_ (°)	d_200_ (Å)	a (Å)	V (Å^3^)
CsPbI_3_	28.27	3.161	6.321	252.7
CsPbI_3_:5Co	28.29	3.154	6.307	251.0
CsPbI_3_:10Co	28.32	3.151	6.301	250.3

## Data Availability

The data that support the findings of this study are available from the corresponding author upon reasonable request.
